# Salivary alpha-amylase activity and its association with early childhood caries and rampant caries experience: a cross-sectional study

**DOI:** 10.3389/fmed.2024.1480139

**Published:** 2025-01-14

**Authors:** Vivek Padmanabhan, Md Sofiqul Islam, Muhammed Mustahsen Rahman, Manjunatha B. K. Goud, Latifa Majed Sulaiman Allay Alshehhi, Hebah Mushref Ahmad Hamed, Sheela Haridas, Dileep Sharma

**Affiliations:** ^1^RAK College of Dental Sciences, Ras Al Khaimah, United Arab Emirates; ^2^RAK College of Medical Sciences, RAK Medical and Health Sciences University, Ras al-Khaimah, United Arab Emirates; ^3^School of Health Sciences, The University of Newcastle, Callaghan, NSW, Australia

**Keywords:** salivary alpha-amylase, early childhood caries, rampant caries, dental caries prevention, ELISA - enzyme-linked immunosorbent assay

## Abstract

**Aims:**

This study aims to evaluate salivary alpha-amylase levels in children diagnosed with Early Childhood Caries (ECC) and Rampant Caries (RC) and compare them to levels in children without ECC or RC. It also examines the relationship between salivary alpha-amylase levels and increased caries activity in the children with ECC or RC.

**Materials and methods:**

A cross-sectional study was conducted at RAK College of Dental Sciences (RAKCODS) with 100 children aged 3–12 years. Participants were divided into ECC and RC groups, each consisting of study and control groups. Salivary alpha-amylase levels were measured using Enzyme-Linked Immunosorbent Assay (ELISA) kits. Statistical analyses, including the Wilcoxon Signed Rank test and Pearson’s correlation coefficient, were performed using SPSS version 29 to compare salivary alpha-amylase levels between groups and examine correlation with severity of dental caries.

**Results:**

The study found that children with ECC and RC had significantly higher mean salivary alpha-amylase levels (16.046 U/mL and 20.62 U/mL, respectively) compared to control groups (5.09 U/mL and 12.70 U/mL). The differences were statistically significant (*p* < 0.0001). Pearson’s correlation coefficients indicated a strong positive correlation between salivary alpha-amylase levels and severity of dental caries in both ECC (*r* = 0.9891, *p* < 0.0001) and RC (*r* = 0.9142, *p* < 0.0001) groups.

**Conclusion:**

The study concludes that elevated salivary alpha-amylase levels, which are statistically significant, are observed in children with ECC and RC. Additionally, it was found that salivary alpha-amylase levels increased with the number of carious lesions. These findings suggest that salivary alpha-amylase could serve as a valuable biomarker for assessing caries risk and guiding preventive strategies.

## 1 Introduction

Dental caries is a widespread and multifactorial oral disease characterized by the progressive destruction of tooth enamel and dentin, primarily driven by interactions between cariogenic bacteria and fermentable carbohydrates (1). This condition remains a major public health concern due to its prevalence and potential to impact overall well-being (1). The etiology of dental caries involves a complex interplay of microbial, dietary, and host factors, including oral hygiene practices, saliva composition, and dietary habits (2). The impact of dental caries extends beyond oral health, affecting essential functions such as chewing and speaking, and influencing quality of life (2, 3). Understanding the contributing factors and progression of dental caries is crucial for developing effective preventive and therapeutic strategies (3).

Early Childhood Caries (ECC) and Rampant Caries (RC) are both severe forms of dental decay but differ in their onset and progression (4). ECC, typically seen in children under 5 years and 11 months of age, primarily affects the upper front teeth and is often linked to prolonged bottle-feeding or frequent consumption of sugary liquids (5). It progresses rapidly, leading to significant decay in a short time (5). Rampant caries, on the other hand, can occur in older children or adults and involves widespread decay across multiple teeth, often triggered by factors like poor oral hygiene, high sugar intake, and insufficient fluoride (6). Both conditions require prompt intervention to prevent extensive damage (4, 6).

Saliva is increasingly recognized as a valuable diagnostic tool due to its ease of collection and rich biochemical profile (7). It contains biomarkers that reflect oral and systemic health, including indicators of dental caries, periodontal disease, and even systemic conditions like diabetes (8). Saliva tests can provide insights into bacterial activity, enzyme levels, and genetic factors, facilitating early detection and monitoring of diseases (9). Additionally, saliva-based diagnostics are non-invasive and can be performed without the need for specialized equipment, making it a practical and cost-effective method for both clinical practice and research (8, 9).

Salivary alpha-amylase is an enzyme that breaks down starches into simpler sugars and is present in saliva (10). Its significance in dental caries lies in its role in carbohydrate digestion and its potential impact on oral health (11). High levels of salivary alpha-amylase might indicate increased starch consumption or altered digestive processes (10). Research suggests that variations in alpha-amylase activity can influence the development of dental caries by affecting the breakdown of dietary carbohydrates, potentially leading to a higher availability of fermentable sugars for cariogenic bacteria (11). Monitoring salivary alpha-amylase levels can thus provide insights into caries risk and dietary habits (10, 12).

There is limited research exploring the correlation between salivary alpha-amylase and dental caries, particularly in the context of Early Childhood Caries (ECC) and Rampant Caries (RC). This study was therefore designed to address this gap in literature. The null hypothesis of this study is that there is no relationship between salivary alpha-amylase levels in children with ECC or RC. The aim of this research is to determine whether a significant relationship exists between salivary alpha-amylase levels in children with and without ECC/RC. Additionally, the study seeks to understand if salivary alpha-amylase levels change with increased caries activity, specifically with a rise in the number of carious lesions, in children diagnosed with ECC and RC.

## 2 Materials and methods

This cross-sectional study was conducted at RAK College of Dental Sciences (RAKCODS), part of RAK Medical and Health Sciences University (RAKMHSU) in Ras Al Khaimah, United Arab Emirates. The goal of this study was to investigate the relationship of salivary Alpha Amylase levels in children diagnosed with Early Childhood Caries (ECC) and Rampant Caries (RC) to those children who did not have active carious lesions. The study also intended to understand if there is a change in Salivary Alpha Amylase levels in children with increasing severity or number of dental caries. Approval for the research was granted by the university’s Research and Ethics committee and the RAK Research and Ethics Committee, Ministry of Health (Proposal/Approval number: RAKMHSU-REC-015-2023/24-UG-D, MOHAP-REC-REF-24/37-2024-UG-D). The samples for the study were collected in the Pediatric Dentistry clinics and the salivary alpha amylase levels were evaluated in the biochemistry lab within the university. The data collection for this study was conducted between November 2023 and May 2024.

Children visiting the RAKCODS pediatric clinics for dental treatment were recruited for the study after obtaining parental consent. Participants, aged 3 to 12 years, were divided into two main groups: Group I (Early Childhood Caries, ECC) and Group II (Rampant Caries, RC), with each group further divided into study and control subgroups. Group I included children under 5 years and 11 months, while Group II included children aged 6 to 12 years. Inclusion criteria for the study groups required at least five active carious lesions, while the control groups consisted of children without active caries. The authors deliberately selected children with minimum of five active carious lesions in the study group to ensure a structured and homogeneous sample, thereby minimizing the possibility of chance occurrences in the study’s findings. Children with a history of medications or hospitalizations that could influence the study variables, or those who did not provide consent, were excluded.

### 2.1 Sample size calculation

The university pediatric dentistry clinic, operating four evenings weekly, receives an average of 3–4 new patients under 5 years 11 months (ECC group) and 4–5 new patients aged 6–12 years (RC group) per week. This allowed for an estimated enrollment of 60–70 ECC children and 70–80 RC children, both with and without dental caries. Using the Raosoft Sample Size Calculator with a 5% margin of error and 90% confidence level, sample size of 100 for the study was deemed optimal, with 25 participants each in study and control groups. Basic demographic data were collected, and the oral screening sheet, utilized as part of the institution’s community services, was employed to assess oral health. This included evaluating the child’s caries status and recording findings such as the DMFT/dmft index. The children were divided into study and control groups based on the presence or absence of dental caries. Twenty-five children were included in the study group with at least five active carious lesions of each ECC and RC. Twenty-five children with no active dental carious lesions were included into the control groups of each ECC and RC. The children enrolled in the study typically visited the clinics between 3: 30 p.m. to 6: 30 p.m., corresponding to the regular operating hours of the pediatric dentistry clinic. After obtaining consent from both the children and their parents, appointments were scheduled. Prior to their visit, the participants were requested to refrain from eating or drinking anything for a minimum of 2 h. Additionally, they were asked to rinse their mouth before saliva collection. The principal investigator provided comprehensive training to all co-investigators regarding data recording and dental examination procedures for children. To prevent any potential bias in recordings, one co-investigator was responsible for data recording during clinic visits, while another was trained to conduct intraoral examinations and saliva sample collection to avoid examiner bias. For salivary alpha amylase estimation, unstimulated saliva samples were collected after obtaining consent from both guardians and children, where participants were requested to slightly bend their head forward, relax, and passively drool accumulated saliva for 5 min into a graduated tube using the Coachman’s Position (8); the saliva samples were then stored at 4°C in an icebox and promptly sent to a laboratory within 20 min for examination of cortisol levels using an Enzyme-Linked Immunosorbent Assay (ELISA) kit (13).

### 2.2 Statistical analysis

The data were analyzed using statistical software SPSS version 29 (IBM Corp. Released 2022. IBM SPSS Statistics for Windows, Version 29.0. Armonk, NY: IBM Corp). The analysis aimed to compare the average salivary Alpha Amylase levels between the study and control groups for both ECC and RC groups. The Wilcoxon Signed Rank test, a non-parametric test used to determine significant differences between two related groups, was employed for this comparison. Furthermore, the analysis sought to examine the correlations between Salivary Alpha Amylase levels and dental caries using Pearson’s correlation coefficient. This coefficient measures the strength and direction of the linear relationship between two continuous variables, helping to determine the association between salivary Alpha Amylase levels and severity of dental caries. A *p*-value threshold of less than 0.05 was set to determine statistical significance. By performing these statistical analyses, the authors aimed to identify potential differences in salivary Alpha Amylase levels between the study and control groups and explore the relationship between these levels and dental caries. These findings could provide valuable insights into the influence of salivary Alpha Amylase on dental health and guide future research or interventions in this field.

## 3 Results

**Figure 1:**
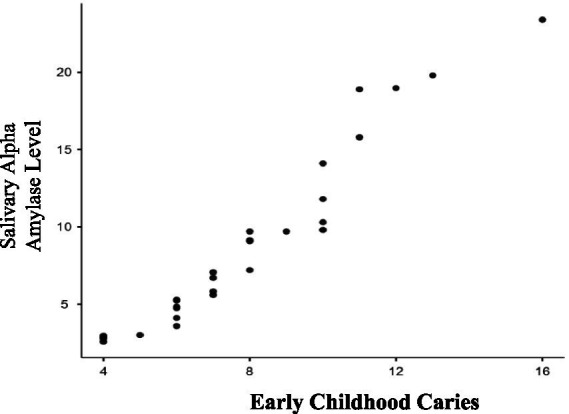
Correlation between severity of early childhood caries and salivary alpha amylase levels. As the severity of early childhood caries (dmft/DMFT) increases as seen in the study group; the salivary alpha amylase levels also increase. There is a strong positive correlation (0.9891) which is statistically significant (*p* ≤ 0.0001).

In this study, evaluated 100 children visiting RAKCODS dental clinics for dental screening and treatment. Both the study and control groups for ECC (Early Childhood Caries) and RC (Rampant Caries) comprised 25 children each, adhering to age criteria: ECC for children under 5 years and 11 months and RC for those between 6 and 12 years. Key variables collected for each group were dental caries status and salivary alpha-amylase levels, measured using an ELISA kit. Table 1 presents results for Group 1 (ECC), comparing mean salivary alpha-amylase levels between the study group (children with active carious lesions) and the control group (children without active carious lesions). The study group had mean levels of 16.046 U/mL, while the control group had 5.09 U/mL. The difference was statistically significant with a *p*-value of less than 0.0001 (*p* < 0.0001). Additionally, Pearson’s correlation coefficient of 0.9891 (*p* < 0.0001) indicated a strong positive correlation between salivary alpha-amylase levels and severity of ECC (increase in number of dental caries lesions) (Figure 1).

**Table 1:** Comparison of mean salivary alpha amylase levels between study and control group of children with early childhood caries.

Variable	Group	Mean	Median	SD	*p* value
Age	Control	4.804	4.67	0.918	
	Study	4.644	4.67	0.44	
SAA (U/ml)	Control	5.09	4.9	1.055	<0.0001
	Study	16.046	13.4	5.586	

The mean salivary alpha amylase levels of study group (16.046 U/mL) are significantly higher than those of control group (5.09 U/mL) and the results are statistically significant (*P* value < 0.0001).

**Figure 2:**
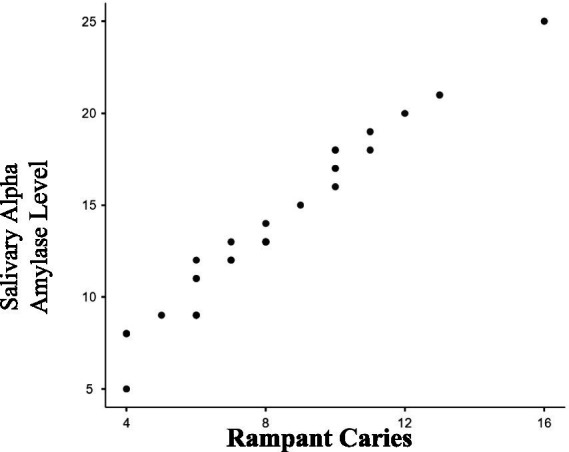
Correlation between severity of rampant caries and salivary alpha amylase levels. As the severity of rampant caries (dmft/DMFT) increases as seen in the study group; the salivary alpha amylase levels also increase. There is a strong positive correlation (0.9142) which is statistically significant (*p* ≤ 0.0001).

Table 2 presents results for Group II (RC), also comparing mean salivary alpha-amylase levels between the study and control groups. The study group had mean levels of 20.62 U/mL, compared to 12.70 U/mL in the control group, with a statistically significant *p*-value of less than 0.0001 (*p* < 0.0001). The Pearson’s correlation coefficient of 0.9142 (p < 0.0001) showed a strong positive correlation between salivary alpha-amylase levels and severity of RC (Figure 2). Overall, the study indicates a statistically significant relationship between the study and control groups of ECC, RC and Salivary alpha amylase levels. The study also reflects a positive association between salivary alpha-amylase levels and increasing severity of ECC and RC as noted in the study groups (ECC and RC).

**Table 2:** Comparison of mean salivary alpha amylase levels between study and control group of children with rampant caries.

Variable	Group	Mean	Median	SD	*p* value
Age	Control	9.56	8.92	2.279	
	Study	8.226	8.17	1.431	
SAA (U/ml)	Control	12.7	13.2	2.681	<0.0001
	Study	20.62	21	4.916	

The mean salivary alpha amylase levels of study group (20.62 U/mL) are significantly higher than those of control group (12.7 U/mL) and the results are statistically significant (*p* value < 0.0001).

## 4 Discussion

The primary aim of this study was to examine the relationship between salivary alpha-amylase levels and the presence of Early Childhood Caries (ECC) and Rampant Caries (RC) in children. Additionally, the study aimed to determine if severity of dental caries altered the salivary alpha-amylase levels. Specifically, the study sought to explore if an increase in the number of active carious lesions was associated with changes in salivary alpha-amylase levels. By examining these interactions, this research sought to provide valuable insights that could guide the development of improved oral health strategies and interventions for children. Salivary alpha-amylase (sAA) plays a dual role in oral health, with both protective and potentially harmful effects. Its protective functions include antimicrobial properties, buffering acids, and supporting remineralization, particularly by enhancing salivary flow and reducing bacterial colonization under normal conditions (11). However, in high-carbohydrate diets or poorly maintained oral environments, sAA can facilitate acid production and bacterial adherence, contributing to caries progression (12). Elevated sAA levels may also reflect an immune response to bacterial activity or stress. Overall, its impact on dental health depends on the balance between these protective mechanisms and environmental factors (12, 13).

In children under 5 years and 11 months with Early Childhood Caries (ECC), elevated salivary alpha-amylase levels as seen in the current study may reflect a protective physiological response. This increase could result from the activation of salivary glands, such as the parotid, submandibular, and sublingual glands, as part of an immune mechanism to regulate bacterial growth (13–15). While alpha amylase promotes acidogenesis by binding to oral streptococci and dental tissues, it may also contribute to acid neutralization and support remineralization within the biofilm (16). Younger children’s underdeveloped motor skills and poor oral hygiene lead to greater plaque retention and bacterial colonization, potentially triggering alpha-amylase production as an adaptive response to persistent caries (14). This aligns with evidence that stress-induced salivary biomarker elevation may function as a defense mechanism (15, 17). Further investigation including the contribution of confounding factors like diet history and oral hygiene practices is needed to better understand the complex interplay between salivary alpha-amylase levels, bacterial activity, and the immune response in ECC. In children of the age group 6–12 years belonging to the Rampant Caries (RC) group, similar elevated salivary alpha-amylase levels were observed. Although dietary history was not recorded in these children, a possible explanation for the increased alpha-amylase production could be diets high in carbohydrates, which are typical for this age group. Such diets may stimulate alpha-amylase activity, facilitating the bacterial fermentation of starches into acids (18–22). Combined with poor oral hygiene practices, this creates a persistent acidic environment that exacerbates caries progression (23, 24). Studies reported worldwide have similarly reported elevated salivary alpha-amylase in older children with severe caries (24–28). However, older children’s improved motor skills compared to those with ECC may reduce plaque retention to some extent (29–31). Despite these age-related differences, the chronic presence of caries may be a reason which stimulates the salivary glands to produce higher levels of alpha-amylase, potentially as an immune response to control bacterial growth, perpetuating a cycle of increased acid production and caries development (17, 31–33). This study, while providing valuable insights into the relationship between salivary alpha-amylase and dental caries, has certain limitations. The relatively small sample size limits the generalizability of the findings. Additionally, confounding factors such as dietary habits, oral hygiene practices, stress levels were not extensively controlled, which may have influenced the results. Some inconsistencies with studies reporting no significant or even inverse relationships between salivary alpha-amylase and caries highlight the need for further research. Future studies should include larger, more diverse populations and investigate a broader range of biochemical and clinical parameters. Longitudinal research designs could also help clarify the causal role of salivary alpha-amylase in caries progression, ultimately contributing to more effective oral health strategies for children.

## 5 Conclusion

In conclusion, this study underscores the significant relationship between Early Childhood Caries (ECC), Rampant Caries (RC), and salivary alpha-amylase levels. The results indicate that salivary alpha-amylase levels are elevated in children with ECC and RC, and their levels increase with the severity of dental caries, whether in ECC or RC cases. These findings highlight the importance of implementing intervention strategies aimed at reducing the factors contributing to dental caries in children, which, in turn, could help lower salivary biomarker levels, including salivary alpha-amylase.

## 6 Recommendations

For General Dentists: Integrating salivary alpha-amylase evaluation into routine dental check-ups can help identify children at higher risk for caries, allowing for personalized preventive strategies. Emphasizing dietary counseling is also crucial, educating patients and families about the impact of carbohydrate consumption on dental health while promoting healthier dietary choices to reduce caries risk. Strengthening oral hygiene education, particularly around effective brushing techniques and proper fluoride use, is key to prevention. However, concerns about the long-term effects of fluoride ingestion have led to increased interest in fluoride-free toothpastes (31). Biomimetic agents like hydroxyapatite, casein phosphopeptide-amorphous calcium phosphate, and calcium sodium phosphosilicate show promise for improving oral health (31). Of these, fluoride-free hydroxyapatite stands out as an effective option, though further studies are needed to identify the best ingredients for tailored preventive care (34).

For Pediatric Dentists: Focus on early detection by implementing diagnostic measures for ECC and RC, including the monitoring of salivary alpha-amylase levels, especially in young children with limited motor skills and oral hygiene abilities. Develop specialized intervention programs for children with elevated alpha-amylase levels, which may involve increased frequency of dental visits, customized preventive treatments, and detailed monitoring of dietary and oral hygiene practices. Furthermore, support and participate in ongoing research to deepen the understanding of salivary alpha-amylase’s role in dental caries, as more comprehensive studies are needed to refine and improve caries prevention strategies.

By addressing these recommendations, dental professionals can improve their management and prevention of caries in children, ultimately leading to better oral health outcomes.

## Data Availability

The original contributions presented in the study are included in the article/supplementary material, further inquiries can be directed to the corresponding author/s.
